# Perspectives on Strengthening Cancer Research and Control in Latin America Through Partnerships and Diplomacy: Experience of the National Cancer Institute’s Center for Global Health

**DOI:** 10.1200/JGO.17.00149

**Published:** 2018-02-22

**Authors:** Silvina Frech, Catherine A. Muha, Lisa M. Stevens, Edward L. Trimble, Roxanne Brew, Doug Puricelli Perin, Silvana Luciani, Alejandro Mohar, Marion Piñeros, Tatiana Vidaurre, Douglas R. Morgan, Ernest T. Hawk, Kathleen M. Schmeler, Lewis E. Foxhall, Cristina Rabadan-Diehl, Denise Duran, Melissa Rendler-Garcia, Eduardo L. Cazap, Luiz Santini, Walter Zoss, Lucia B. Delgado, Paul C. Pearlman, Leslie Given, Karin Hohman, Melissa S. Lopez, Brenda Kostelecky

**Affiliations:** **Silvina Frech**, **Catherine A. Muha**, **Lisa M. Stevens**, **Edward L. Trimble**, **Roxanne Brew**, **Doug Puricelli Perin**, **Paul C. Pearlman**, and **Brenda Kostelecky**, National Cancer Institute, Rockville, MD; **Silvana Luciani**, Pan American Health Organization; **Cristina Rabadan-Diehl**, US Department of Health and Human Services; **Denise Duran**, Centers for Disease Control and Prevention, Washington, DC; **Alejandro Mohar**, Instituto Nacional de Cancerología, Mexico City, Mexico; **Marion Piñeros**, International Agency for Research on Cancer, Lyon, France; **Tatiana Vidaurre**, Instituto Nacional de Enfermedades Neoplásicas, Surquillo, Peru; **Douglas R. Morgan**, Vanderbilt-Ingram Cancer Center, Nashville, TN; **Ernest T. Hawk**, **Kathleen M. Schmeler**, **Lewis E. Foxhall**, and **Melissa S. Lopez**, The University of Texas MD Anderson Cancer Center, Houston, TX; **Melissa Rendler-Garcia**, Union for International Cancer Control, Geneva, Switzerland; **Eduardo L. Cazap**, Sociedad Latinoamericana y del Caribe del Oncología Médica, Buenos Aires, Argentina; **Luiz Santini** and **Walter Zoss**, Red de Institutos e Instituciones Nacionales de Cáncer, Sao Paolo, Brazil; **Lucia B. Delgado**, Universidad de la República, Montevideo, Uruguay; and **Leslie Given** and **Karin Hohman**, Strategic Health Concepts, Arvada, CO.

## Abstract

According to the Pan American Health Organization, noncommunicable diseases, including cancer, are the leading causes of preventable and premature death in the Americas. Governments and health care systems in Latin America face numerous challenges as a result of increasing morbidity and mortality from cancer. Multiple international organizations have recognized the need for collaborative action on and technical support for cancer research and control in Latin America. The Center for Global Health at the US National Cancer Institute (NCI-CGH) is one entity among many that are working in the region and has sought to develop a strategy for working in Latin America that draws on and expands the collaborative potential of engaged, skilled, and diverse partners. NCI-CGH has worked toward developing and implementing initiatives in collaboration with global partners that share the common objectives of building a global cancer research community and translating research results into evidence-informed policy and practice. Both objectives are complementary and synergistic and are additionally supported by an overarching strategic framework that is focused on partnerships and science diplomacy. This work highlights the overall strategy for NCI-CGH engagement in Latin America through partnerships and diplomacy, and highlights selected collaborative efforts that are aimed at improving cancer outcomes in the region.

## INTRODUCTION

Globally, including in Latin America, low- and middle-income countries (LMICs) are experiencing an epidemiologic transition from infectious diseases to cancer and chronic diseases. In fact, LMICs now account for two thirds of the worldwide burden of noncommunicable disease (NCD).^1^ Enhanced infectious disease control, improvements in maternal-child health, and greater access to health services are global health successes and have contributed to the growth and aging of LMIC populations. The changing demographics, coupled with rural-to-urban transitions, environmental exposures, and the adoption of unhealthy lifestyles—for example, obesity, limited physical activity, tobacco use, and alcohol consumption—have resulted in LMICs reaching an NCD tipping point. It has been predicted that Latin American countries will experience additional marked increases in the number of cancer cases in the near term. The International Agency for Research on Cancer (IARC) has estimated that the cancer burden in the Latin America region will increase by 66%, from 1.1 million new cancer cases and 500,000 cancer deaths in 2012 to more than 1.6 million new cancer cases and 1 million cancer deaths by 2030.^2,3^

Recent studies have indicated that the leading incident cancers in Latin America among males are prostate, lung, colorectal, and stomach cancer, and breast, cervix, colorectal, and stomach cancer among females.^2,4^ The highest number of cancer deaths are a result of stomach, prostate, lung, and colorectal cancer for males, and breast, cervix, stomach, and lung cancer for females. Variability in disease burden by cancer site is found between countries. Countries with a high or very high human development index have a higher burden of prostate and breast cancer, whereas countries with a medium human development index have higher burdens of stomach and cervical cancers. Available cancer incidence data must be interpreted with caution, as the best-quality data covers less than 10% of the region’s population.^5^ This highlights the importance of data that are generated by population-based cancer registries (PBCRs) as a crucial element for cancer control planning. Strengthening registry systems is critical to better guide cancer policy and programs on the basis of high-quality and current evidence. Furthermore, surveillance data allow program managers to evaluate the impact of cancer control actions and to perform research to better understand existing patterns.^6^

Whereas most countries in the region have made advances in cancer registration^7^ and in preventing and controlling cancer and other NCDs, these diseases continue to present significant public health challenges that often reflect country-specific socioeconomic conditions and health resource allocation.^8^ Two publications of The Lancet Oncology Commission on Latin America report in detail on the status of cancer control and associated challenges that face the region’s countries,^9,10^ including inequities in health service access, limited financial resources that leads to limited infrastructure and medicine access, highly heterogeneous populations from indigenous peoples to densely populated cities, and inadequate numbers of trained health professionals. Some countries have demonstrated a strong political commitment to universal health coverage and have made substantial improvements; however, there is still a need to implement—and monitor accordingly—high-impact interventions to reduce modifiable risk factors, increase vaccination rates for vaccine-preventable cancers, strengthen programs for appropriate cancer screening and early diagnosis, and improve affordability, access, and the quality of cancer care.^11^

Many countries in Latin America are developing or have recently developed national cancer control plans (NCCPs^12^), national site-specific cancer programs (eg, breast, cervix, and colorectal cancer), and national NCD plans. The Lancet Oncology Commission, in 2015, recommended that the implementation and success of the newly signed NCCPs in Latin America should be monitored and that the process should be done in cooperation with international organizations.^10^ According to the IARC World Cancer Report, NCCPs present an opportunity for countries to implement effective change at the population level on an ongoing, adaptable basis by using a variety of interventions that can be adjusted to contextual, sociocultural, and financial means of even the poorest nations.^13^ In fact, that countries develop national NCD plans by 2015 is one of the time-bound global commitments for NCDs established by the United Nations.^14^ Furthermore, the World Health Assembly, in 2017, approved a cancer resolution that reiterates the value of NCCPs and specifically urges countries to collect high-quality data to guide their planning.^15^

Partnerships are the cornerstone of international cooperation and technical assistance.^16^ A successful, sustainable development agenda requires partnerships between governments, the private sector, and civil society. Inclusive partnerships build on shared vision and goals at the global, regional, national, and local levels. Since its creation in 2011^17^ and designation as a WHO collaborating center in 2016, the Center for Global Health at the US National Cancer Institute (NCI-CGH) has leveraged opportunities for multisectoral partnerships and science diplomacy in support of initiatives that are aimed at strengthening cancer research and control globally, including in Latin America. NCI-CGH has taken a lead role in some of these initiatives while providing support for other efforts led by partners. The initiatives outlined in this work include the efforts of multiple national and international partnerships, many of which have built on preexisting initiatives and been sustained over multiple years.

## RESEARCH FUNDING AND NETWORKS

Working closely with other NCI divisions, NIH institutes, and NCI-designated cancer centers, NCI-CGH has developed and participated in research programs and initiatives that both promote cancer research and control and develop research underpinnings and networks to facilitate collaboration and increase efforts to address the global cancer burden, with emphasis on LMICs.^18^

Since 2009, the NCI has supported the Latin America Cancer Research Network (LACRN), a coalition of more than 40 research institutions in Argentina, Brazil, Chile, Mexico, and Uruguay that aimed to strengthen translational cancer research capacity in Latin America. The initial project for LACRN was an observational breast cancer molecular profiling study.^19^ Building on the experience and relationships established through LACRN with US, international, and Latin American partners, NCI-CGH has additionally expanded efforts to support cancer research by strengthening this scientific community.

NCI-CGH has established two recent funding initiatives—2014 to 2015 and 2015 to 2017—to support NCI-designated cancer centers and stimulate and strengthen cancer control research partnerships between investigators at US and LMIC research institutions. Two awarded programs from these initiatives are led by Vanderbilt-Ingram Cancer Center, which partnered with health ministries and universities in the Central America Four (CA-4) that consists of Guatemala, Honduras, El Salvador, and Nicaragua. The CA-4 is a geopolitical entity that has had open borders since 2006 and that covers a population of 36 million. Components of the programs include building PBCR technical capacity and gastric cancer molecular profiling capability in the CA-4,^20^ as well as executing the El Salvador national gastric cancer incidence study. The research awards also funded the first CA-4 Cancer Bioinformatics Conference—San Salvador, October 2014—with additional support from the Union for International Cancer Control (UICC) and IARC Global Initiative for Cancer Registry Development (GICR),^21^ and laid the foundation for planning the Central America Leadership Forum described in the Cancer Control Planning section of this article. NCI-CGH funding in this area has provided a foundation for long-term programs to support high-quality scientific research that can ultimately benefit both the US population and countries around the world.

NCI-CGH has also sought to strengthen partner-led networks for cancer research between NCI-designated cancer centers and Latin American institutions. For example, The University of Texas MD Anderson Cancer Center (MDACC), in collaboration with their Sister Institution Network in Latin America, are performing multicenter research projects, such as the ConCerv study, which evaluates the safety and efficacy of conservative surgery for early-stage cervical cancer. This study is being carried out at sites in Colombia, Peru, Brazil, Mexico, and Argentina, and has already created a framework for performing clinical trials across countries to improve research capacity and in-country data to inform changes in local guidelines and policies.^22^ If successful, this trial will provide necessary data to change the standard of care for the surgical management of early-stage cervical cancer in the United States and Latin America. This network is also involved in evaluating low-cost technologies for point-of-care diagnostics for cervical cancer, with ongoing studies related to the high-resolution microendoscope developed by Rice University.^23,24^ NCI-supported studies that are evaluating the high-resolution microendoscope are ongoing in El Salvador and Brazil and could provide an alternative, low-cost method for the screening and diagnosis of cervical cancer.

## TRAINING IN CANCER CONTROL AND RESEARCH

Training and education are important components of building a global cancer research community. NCI-CGH provides support for highly qualified health professionals and scientists from the region and around the world to attend the annual NCI Summer Curriculum in Cancer Prevention.^25^ A recent evaluation of the program determined that course participants from LMICs reported increases in knowledge and skills (98% of respondents), and an enhanced ability to apply the knowledge gained to cancer control activities (97% of respondents) in their home countries.^26^ Furthermore, 67% of respondents reported having published or presented information on cancer prevention directly on the basis of on their experience in the course, and 43% reported receiving grants. The course content is of great interest to other countries, and discussions between NCI-CGH, National Cancer Institute of Mexico, and MDACC have focused on the development and testing of a modular, electronic, culturally tailored curriculum in cancer prevention and control for use in Latin America. The modified course would aim to advance the public’s and professionals’ knowledge and practice of research directly related to evidence-based cancer prevention.

An example of an initiative to provide training and promote the practical use of evidence focuses on implementation research, a nascent discipline in Latin America. The Introduction to Cancer Program Planning and Implementation Research Workshop, co-organized by the National Cancer Institute of Argentina (INC), NCI-CGH, and the NCI Division of Cancer Control and Population Sciences, was held in Buenos Aires, Argentina, in November 2014. This was the first workshop on this topic in the region, with expert faculty from the United States and Latin America. After the workshop, INC has continued to invest in implementation research training and has developed and funded implementation research grants, which has demonstrated the importance of international collaboration to promote new science avenues in the region.

Involvement in partner-led initiatives to improve cancer control and research training has complemented the initiatives of NCI-CGH in several ways. Project ECHO (Extension for Community Healthcare Outcomes) is one such example of a partner-led effort and has recently been implemented as a strategy to improve cancer care by connecting interdisciplinary specialist teams in high-resource areas with community primary care providers in low-resource regions to increase clinical capacity. Project ECHO is a powerful, low-cost, high-impact telementoring initiative that aims to improve both capacity and access to specialty care for rural and underserved populations.^27^ Specialists use videoconferencing to comanage patients and share expertise via mentoring, guidance, feedback, and didactic education. MDACC has adapted the Project ECHO model to educate and support local providers in the management of cervical dysplasia and invasive cervical cancer in both Latin America and Texas along the Texas-Mexico border, where more than 90% of the population is Hispanic.^28^ This approach has enabled clinicians in medically underserved areas to develop the skills, confidence, and knowledge to treat patients with common, complex diseases in their own communities, thereby reducing travel costs, wait times, and avoidable complications. NCI-CGH has supported network building within the Cancer Control Leadership Forums (CCLFs) described in the secton on Cancer Control Planning to link MDACC’s robust telementoring program to more researchers, clinicians, and policymakers. This network building aims to additionally strengthen the capacity for research and evidence-based care and to develop a network of countries that learn from each other about cervical cancer challenges and solutions in the region.^29^

GICR—led by IARC, with technical support from NCI-CGH—is a partner-led initiative to support important improvements in cancer registry development and training. PBCRs are essential for effectively informing the planning, implementation, and evaluation of comprehensive NCCPs and are thus of interest to national stakeholders and supporting organizations.^6,30^ Despite the existence of numerous PBCRs in the region and efforts in cancer registration, few registries in Latin America have met data quality standards—comparability, completeness, and validity—to be included in Volume X of IARC’s serial publication, Cancer Incidence in Five Continents. The PBCRs included in the Cancer Incidence in Five Continents publication cover only 8% of the population living in Latin America,^5^ which highlights the urgent need for governments in the region to strengthen PBCRs. To address the observed inequities in this and other less-developed regions, IARC launched GICR with the goal of increasing data to adequately inform cancer prevention and control, permitting an improved ability to plan and evaluate cancer programs in LMICs.^21^ The IARC-led GICR is a unique partnership that brings together several organizations, including the International Atomic Energy Agency, US Centers for Disease Control and Prevention (CDC), UICC, the American Cancer Society, the International Association of Cancer Registries, and NCI. Five regional hubs have been established within a unified framework to provide training and support and to foster networks for cancer registries in all regions of the world. The GICR Latin America Hub, with IARC, is coordinated by INC Argentina, and collaborates with centers in Brazil, Uruguay, and Colombia. Among its functions, the Latin America Hub provides technical assistance and support, organizes education and training, promotes networking and communications (eg, virtual forum), and supports cancer research and control (eg, publications) in the region.^7^

## CANCER CONTROL PLANNING

Cancer control planning has also been one of the key programmatic areas that NCI-CGH has sought to strengthen as an avenue to improve the translation of research results into policy and practice. An NCCP, as defined by the WHO, is a strategic plan designed to reduce the number of new cancer cases and cancer-related deaths, as well as to improve the quality of life of patients with cancer, through the systematic and equitable implementation of evidence-based strategies for prevention, early detection, diagnosis, treatment, and palliative care, making the best use of available resources in the country.^31,32^

In 2006, the American Cancer Society, CDC, NCI, and the International Union Against Cancer (now UICC) began a project to support cancer control planning in Latin America that adapted the model of the Comprehensive Cancer Control Leadership Institute that had been successfully used to develop state cancer plans in the United States.^33^ The adapted CCLFs were initially held in Mexico and Brazil in 2006 and 2007, respectively, with the goal of providing technical support to countries for the development of strategies to implement their NCCPs.^34^

Upon the formation of NCI-CGH several years later, the CCLF program was revitalized, and CCLFs in Latin America were held in 2015 and 2016 in collaboration with numerous international organizations that comprise the International Cancer Control Partnership (ICCP).^12,35^ The goals of the CCLF are to increase a country’s knowledge of initiating or enhancing comprehensive, evidence-based cancer control planning on the basis of a multisectoral approach; exchange challenges, successes, and best practices, and promote multidirectional learning among Latin American countries and between Latin American and US participants; and increase awareness among country stakeholders of the importance of incorporating evidence in NCCPs. Latin American subregions were engaged in both the 2015 and 2016 CCLFs. The 2015 CCLF took place in Cancun, Mexico, with multisector teams of participants from Argentina, Brazil, Chile, Colombia, Mexico, Peru, and Uruguay, and individual representatives from Ecuador, El Salvador, Guatemala, Honduras, Panama, and Paraguay. The 2016 CCLF was held in Antigua, Guatemala, and included multisector country delegations from Costa Rica, the Dominican Republic, El Salvador, Guatemala, Honduras, Nicaragua, and Panama, and an individual representative from Belize.

Collaboration on the renewed CCLF has included coordination with global organizations that work on cancer research and control in Latin America to avoid a duplication of efforts and to efficiently use limited technical assistance resources to ultimately support better outcomes in the region. The 2015 and 2016 CCLFs for the Latin American region included the following ICCP partners: The Pan American Health Organization (PAHO)/WHO, MDACC, UICC, CDC, IARC/GICR, the Breast Cancer Initiative 2.5, the Latin American and Caribbean Society of Medical Oncology, and the Network of Latin American National Cancer Institutes of the Union of South American Nations (RINC/UNASUR). Additional partners included the National Cancer Institute of Mexico, the Vanderbilt-Ingram Cancer Center, the University of Miami, the Dana-Farber Cancer Center, and the Office of Global Affairs at the US Department of Health and Human Services. CCLF partners supported a range of activities, from hosting to content development, and from country team facilitation to on-site and follow-up technical assistance.

The CCLF program is tailored to meet the needs of participating countries and their different stages of NCCP development. Country delegations were represented by ministry of health–nominated professionals and representatives from leading stakeholder organizations, including academic institutions and civil society groups. The CCLFs presented an opportunity for countries to learn from other countries with similar challenges, as well as from international experts, how best to improve their cancer plans and programs. The forum format was centered on interactive discussions, which provided country delegations ample opportunity to share experiences and lessons learned with a mixture of didactic modules on core aspects of cancer control planning, special topic presentations (eg, cost-effectiveness studies, the role of academic research, and advocates in planning), roundtable discussions, and country team action planning. By the end of the 3-day CCLF, a detailed 12-month action plan was developed by each country to move their respective NCCP efforts forward. Follow-up engagement with each country delegation was built into the program to support sustained technical assistance for 12 months after the forum. The most common strategic priorities included in the action plans that were developed at the CCLFs were revisions of draft NCCPs, the strengthening resource mobilization, and the development and/or strengthening of PBCRs. Central American countries prioritized improvements in cervical and stomach cancer control programs, whereas South American countries and Mexico focused on strengthening national cancer control partnerships.

Outcomes from the CCLF included improved knowledge and skills in specific areas of cancer control planning, for which average self-reported knowledge improvement ratings ranged from 2.6 to 2.9 on a 3.0 scale for specific questions on the development of NCCPs, best practices in partnerships, and the use of data to guide NCCP efforts. Given the individualized nature of country action plans and the long timeframe for NCCP development and implementation, the measurement of longer-term outcomes was more challenging. Examples of reported outcomes after 12 months include the completion of the Chilean national strategy for cancer control—now available for public review—and the first multidisciplinary forum on cancer in Argentina, held in October 2016, to bring together government officials, academics, the private sector, professional societies, and patient advocacy groups. There were also several examples of additional tailored follow-up by partners, including the following: CDC provided virtual training to the Uruguay team to develop their NCCP’s evaluation plan; MDACC engaged in research and training collaborations with Latin American groups that participated in the forums, including the Project ECHO expansion; and IARC/GICR provided support for training in statistical data analysis to evaluate the impact of specific cancer control measures.

RINC is an important regional leader in cancer control planning and an ICCP partner that collaborated on the CCLF program. Created in 2011, RINC is an UNASUR-Salud institution composed of representatives of national cancer institutes and/or ministries of health that are responsible for cancer control planning in Latin America. As an intergovernmental platform for technical assistance that is based on south–south cooperation, RINC has managed to achieve strong political outreach in a short time by effectively promoting the exchange of best practices, fostering the alignment of national strategies, and sharing knowledge.^36^ NCI and other ICCP partners—for example, UICC, IARC, and PAHO/WHO—coordinate with RINC to provide technical support for common regional priorities and activities, including the implementation of high-priority programs (eg, regional technical assistance) and the organization of training workshops in the region (eg, cancer registration, pathology, and biobanking). The technical assistance initiative carried out by RINC in 2016—South America Free of Cervical Cancer—was approved by ministers of health in Uruguay in November 2015 with the goal of preventing the emergence of new cervical cancer cases and stopping unnecessary deaths.^37^ The RINC initiative dovetailed well with the CCLF and other joint collaborations and is an important milestone for the region as it demonstrates the benefits of cooperation between countries to tackle the cancer burden in Latin America.

## SCIENCE DIPLOMACY

Science diplomacy has the overarching goal of using science and scientific cooperation to build bridges between countries and to promote international understanding and prosperity as essential elements of foreign policy.^38^ Partnerships that include scientists, health care providers, policy experts, and diplomats can effectively advance global science for the benefit of all peoples.^38^ Such benefits can include advancing health science through international cooperation, expanding knowledge bases to enable improvements to health and well-being, and improved international relations. As such, NCI-CGH has sought to work with Latin American country embassies in Washington, DC; health attachés from the US Department of Health and Human Services posted in Brazil and Mexico; US embassies throughout Latin America; and multilateral organizations, such as PAHO, in addition to traditional academic and civil society partners.

The 2015 agreement established between Peru’s Instituto Nacional de Enfermedades Neoplasicas (INEN) and the NCI is an example of how science diplomacy has been used to help build a stronger global cancer research community. The agreement aims to advance collaboration in areas of mutual interest, including collaborative research projects and human resources training, through technical workshops on cancer research and control. The establishment of the agreement required the initial involvement of US and Peruvian diplomatic professionals to come to consensus on the appropriate framework for collaboration supported at the highest levels of government. Since the agreement was established, a multisectoral delegation from Peru participated in the CCLF held in 2015, and INEN leadership participated in global cancer control advocacy activities—for example, by contributing their expertise to the recent revision of the WHO list of essential medicines. High-level collaboration was additionally strengthened during then-President Obama’s visit to Peru in 2015. More recently, NCI and INEN co-organized a workshop—Accelerating Action on HPV and Cervical Cancer Prevention and Control: Implementing Policy Recommendations—in Lima, Peru, in 2016, as part of the multilateral Asia–Pacific Economic Cooperation (APEC) forum. The workshop led to the development of an implementation roadmap that includes the creation of an electronic knowledge library, a community of practice, and pilot projects to support strengthening evidence-based cervical cancer prevention and control in Asia–Pacific Economic Cooperation economies.

Building on the relationships established between the United States and Peru, a Cancer Control in Latin America roundtable was hosted by the then-Peruvian Ambassador to the United States in 2016 in Washington, DC. The goal of the roundtable was to begin a regional dialogue on strategies for strengthening cancer research and control. The dialogue raised awareness among high-level government officials about the importance of including cancer control in the agendas of country leaders and governments. The roundtable also identified opportunities for embassies to engage with and support scientists from their home countries, including those training in the United States. There was agreement among participants that information sharing is essential to support the efficient use of limited resources, avoid the duplication of efforts, and synergize ongoing investments in specific countries. Embassies can serve as conduits for such information sharing among both government and nongovernment stakeholders in their home countries, as well as among their expatriates located in the United States.

## CONCLUSION

Several partners, including NCI-CGH, have the common goal of strengthening cancer research and control in Latin America. Given the role of the NCI a research agency, NCI-CGH has sought to accomplish this goal primarily by strengthening the cancer research community within the region and between US and Latin American institutions, and by supporting the translation of research results into practice and policy. The strategies used have included targeted initiatives to provide support for collaborative research in Latin America, research funding and network building, training, and evidence-based cancer control planning and implementation. The interplay and complementarity between these three strategies is intended to create a sustained capacity for research for improved human health in the region.

Our experience implementing programs in partnership with diverse stakeholders has provided many tangible benefits. In NCI-funded research projects in Latin America, dedicated academics have donated their own time to provide enhanced mentorship to local researchers to increase local capacity, and have worked with local communities and policymakers to improve the potential of their research to be translated into policy and practice. In training programs, partners have provided financial support for trainees from LMICs and have lent their technical expertise, cultural understanding, and language skills to ensure that knowledge is effectively transferred. Partnership benefits are particularly apparent in cancer control planning, for which many partners are needed to identify the right stakeholders in different countries and from multiple sectors on the basis of their knowledge of the landscape; to provide expertise in areas that span scientific research and practical knowledge on soliciting support for and implementing programs; to contribute to the costs of convening large groups of multisector professionals; and to maintain momentum and focus among large groups of partners.

The challenges that are faced in these partnerships for cancer research and control initiatives include a lack of time on the part of different partners who often have additional responsibilities, differing objectives and priorities between partner organizations, and competition between organizations for resources and credit. In our experience, one strategy that has promoted longer-term engagement is ensuring that partners work on meaningful activities that add value to what the partners could do on their own. Partners who understand clearly why their involvement adds value are more motivated and can justify to their internal organizational leadership the investment in time and resources. Another important strategy is taking the time to come to consensus on program goals and implementation. Although it takes time to engage in several rounds of input and negotiation, an open and transparent process of program development benefits the partners and strengthens the final program.

The impact of partnerships on the promotion of stronger worldwide research capacity has the potential to extend beyond Latin America, as all countries, including the United States, require innovative and affordable solutions to reduce the cancer burden, which carries a substantial human and economic toll. Global partnerships and science diplomacy are cornerstones of the work that NCI-CGH does and have provided remarkable opportunities to improve the cohesion of the global cancer research community and enhance ongoing national cancer control planning efforts to ultimately improve cancer outcomes worldwide. Future efforts in Latin America will continue to focus on current strategic priority areas, with additional concerted efforts being placed on new collaborative opportunities.
